# Effect of maternal obesity on insulin action in male adult offspring rats

**DOI:** 10.1186/1758-5996-7-S1-A127

**Published:** 2015-11-11

**Authors:** Eduardo Kloppel, Yuri Karen Sinzato, Debora Cristina Damasceno, Gustavo Tadeu Volpato, Kleber Eduardo Campos

**Affiliations:** 1 UFMT, Barra do Garças, Brazil

## Background

Obesity is a metabolic disturbance that more affects the population in 21st century. Among these metabolic changes, the glucose intolerance and insulin resistance may be developed by aging and also influence in further generations.

## Objective

to evaluate the secretion and action of endogeous insulin in adult age of rats from a gestational obesity.

## Materials and methods

twelve newborn female Wistar rats were used, and half of them submitted to saline solution administration (control) and the other half were administrated monosodium glutamate solution, 4.0 mg/Kg body weight (obese) in neonatal period. At adult age (90 days of life) these female rats were mated with health male rats and the male offspring were used, divided into two groups: control (CONT, n=29) and obese (OB, n=19), according to its previous dam group. In all adult age (from 3rd to 7th months) the rats were monthly evaluate the Lee Index, water and food intake, 12h-fasting glycemia, oral glucose tolerance test (OGTT) and insulin test tolerance (ITT). In addition, from OGTT Results it was estimated the area under the glycemic curve (AUC). All data were statistically analyzed with 5% significance.

## Results

20% of CONT rats were classified as obese by Lee Index only in 7th month, whereas 100% of OB rats were classified as obese. Moreover, the OB rats showed increasing of food intake at 4th and 7th month and water intake in 4th month. CONT rats presented higher food intake from 5th to 7th months, all compared to 3rd month. When both groups are compared, OB rats presented an increased food intake in months 3 and 4. In both groups the 12h-glycemia were higher only in 7th month. The OGTT data showed a progressive disturbance in glycemic curve, once in the 3rd and 4th months the curves presented a classic design (glycemic timepoints higher in 30′ and decreasing at 60′ and 120′), at 5th month the serum glucose increased at timepoint 30′ in both groups and in 6th month only in OB group. In the last month, the OB group presented higher gycemia in all timepoints in OB rats, and the ITT presented no insulin effect, because all timepoints did not presented glucose changes.

## Conclusion

The gestational obesity has the ability to induce the obese state to next generation, associated with glucose intolerance and insulin tolerance with aging, suggesting lower insulin effects in peripheral tissues.

